# The Impact of Vitrimers on the Industry of the Future: Chemistry, Properties and Sustainable Forward-Looking Applications

**DOI:** 10.3390/polym12081660

**Published:** 2020-07-26

**Authors:** Walter Alabiso, Sandra Schlögl

**Affiliations:** Polymer Competence Center Leoben GmbH, Roseggerstrasse 12, A-8700 Leoben, Austria; walter.alabiso@pccl.at

**Keywords:** self-healing, recyclable, vitrimer, transesterification, industrial applications, covalent adaptable networks, bio-renewable, thermosets

## Abstract

Thermosets are known to be very reliable polymeric materials for high-performance and light-weight applications, due to their retained dimensional stability, chemical inertia and rigidity over a broad range of temperatures. However, once fully cured, they cannot be easily reshaped or reprocessed, thus leaving still unsolved the issues of recycling and the lack of technological flexibility. Vitrimers, introduced by Leibler et al. in 2011, are a valiant step in the direction of bridging the chasm between thermoplastics and thermosets. Owing to their dynamic covalent networks, they can retain mechanical stability and solvent resistance, but can also flow on demand upon heating. More generally, the family of Covalent Adaptable Networks (CANs) is gleaming with astounding potential, thanks to the huge variety of chemistries that may enable bond exchange. Arising from this signature feature, intriguing properties such as self-healing, recyclability and weldability may expand the horizons for thermosets in terms of improved life-span, sustainability and overall enhanced functionality and versatility. In this review, we present a comprehensive overview of the most promising studies featuring CANs and vitrimers specifically, with particular regard for their industrial applications. Investigations into composites and sustainable vitrimers from epoxy-based and elastomeric networks are covered in detail.

## 1. Introduction

It is common knowledge that polymeric materials break down into two rather broad and diverse major classes: thermoplastics and thermosets. The general features of polymeric materials include chemical stability, thermal and electrical insulation, low cost, light weight, as well as a plethora of tunable properties, which make them appealing for applications in the aerospace, automotive, electronics and biomedical sectors [1,2].

**Figure 1:**
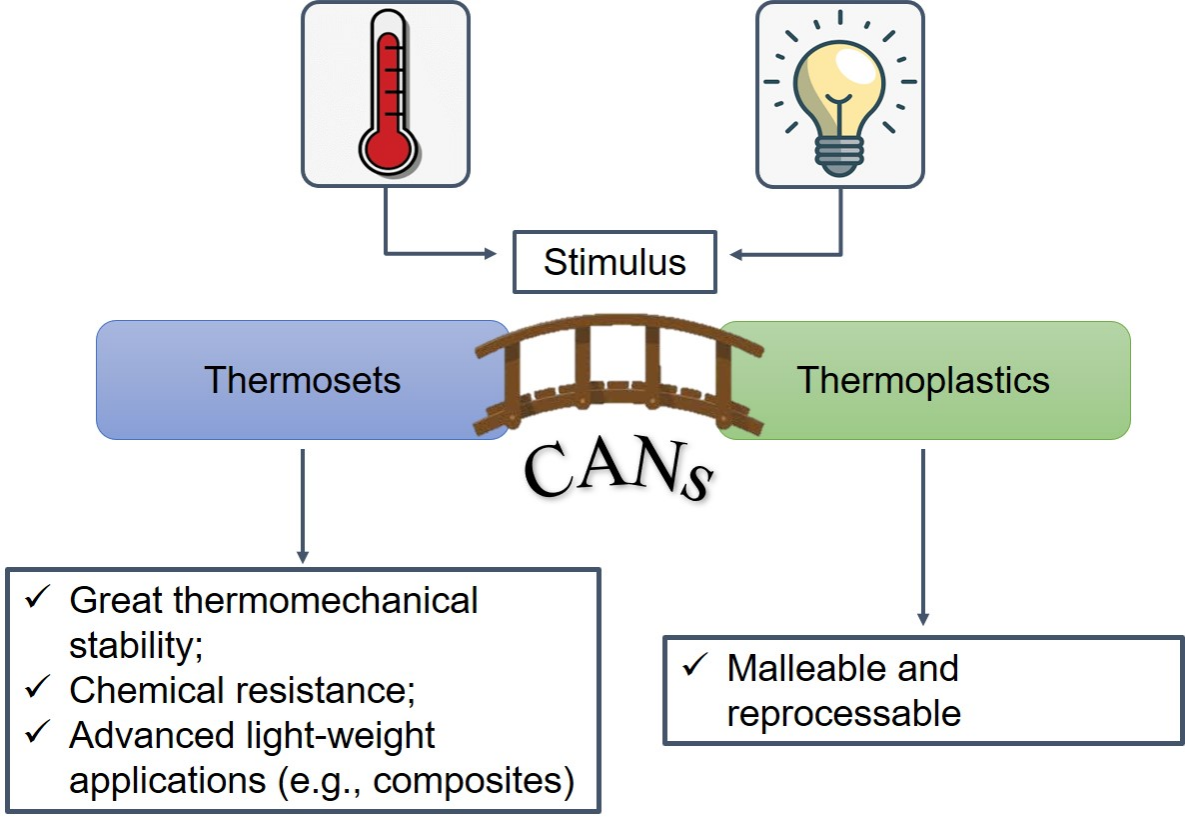
Covalent Adaptable Networks (CANs) as an intermediate bridge between thermosets and thermoplastics.

On the one hand, thermoplastics are typically inherently amenable to recycling, reshaping and easy processing due to the non-covalent nature of the bonds holding their chains together. This enables them to flow like a viscoelastic fluid above a specific temperature (or range thereof) and makes them soluble in a good solvent [3,4]. On the other hand, thermosets are insoluble and unable to flow, due to their cross-linked network. Once their shape is set through the so-called curing process, they are further thermally unprocessable and only swell in a good solvent, often with negligible traces of residual dangling chains being eluted away. However, this characteristic is also advantageous, as it imparts attractive properties such as an extended range of thermo-mechanical stability and chemical, wear and creep resistance [5]. Because of their sturdiness, thermosets are vastly deployed for high performance and light-weight applications (e.g., composite materials), though with major concerns regarding non-recyclability and environmental impact [3,4,6,7,8]. As a side note, one may also include elastomers as a thermosetting polymer subclass. These materials are able to withstand a huge deformation thanks to the entropic nature of their elastic recoil [9]. They are very tough and solvent-resistant materials that have been used for industrial applications for decades, such as vulcanized natural rubber for tires [10] or Polydimethylsiloxane (PDMS) for electrical insulation [11]. Over the past decade, many attempts to bridge the gap between thermosets and thermoplastics have been made (see Figure 1), owing to the strong enticement of relevant industrial applications.

Getting the best of both worlds would ideally result in a material that retains strong mechanical properties over a large range of temperatures and, at the same time, that is prone to sought-after properties like self-healing, recyclability and weldability. The concept of self-healing is rather broad: it spans a multitude of fields (biomedical and aerospace to mention some) and can be potentially applied to polymeric materials, as well as ceramics and metals. Whereas self-healing has the goal to allow the reparation of cracks and defects, possibly spontaneously and seamlessly, recyclability would promote an efficient and sustainable management of the resources. In addition to that, weldability could allow for new design options, with complex geometries and flexible assembly of several structural parts. We claim that the synergistic combination of these properties ultimately leads to versatility, extended life-span and decreased costs. In 2011, Leibler et al. pioneered a ground-breaking novelty in the field of polymer chemistry, which was met with a staggeringly rising interest in the scientific community. The authors rethought the chemistry of cross-linked polymers and proposed Covalent Adaptable Networks (CANs) with silica-like fluidity, vitrimers [12]. Vitrimers are able to readily flow above a characteristic temperature (topology freezing transition temperature, T_v_), while behaving like rigid thermosets below it. In the firstly introduced networks by Leibler et al., this property is a consequence of the thermally activated bond exchange via transesterification [12]. Introducing thermosets to conventional shaping techniques such as injection moulding and extrusion (so far the exclusive prerogative of thermoplastics) would have a tremendous impact on industry. Tuning the speed of bond exchange according to the intended purpose, implementing easy and low-cost formulations, overcoming the natural trade-off between mechanical properties and healing efficiency are in our opinion the major hurdles. After the bright work of Leibler and coworkers, an astounding number of approaches to introduce new chemistries, monomers, catalysts and synthesis strategies have been pursued.

In this review, we aim to thoroughly cover the characteristics of dynamic covalent networks and vitrimers specifically. We would like to offer the reader a comprehensive view of the recently-proposed papers that caught our attention. Inasmuch as the chemistry of such systems has been extensively covered elsewhere (we address the reader to some insightful reviews [3,4,6,7,13,14,15]), we would like to focus our attention on studies that hint at a solid underlying industrial applicability. Therefore, Section 2 and Section 3 below will provide a theoretical background of CANs. In addition to that, Section 4 highlights the most interesting properties stemming from vitrimers, whilst ultimately, Section 5 focuses on relevant concrete perspectives. Because of the high attention industries currently direct toward sustainability and novel bio-renewable materials, we decided to highlight vitrimers in environmentally-friendly applications as well. To the best of our knowledge, such a pragmatic slant with emphasis on a vast and organic collection of industrially-relevant and sustainable vitrimeric materials has not been proposed yet.

## 2. Theoretical Background of Dynamic Covalent Networks and Vitrimers

### 2.1. Associative and Dissociative Covalent Adaptable Networks

Dynamic bonds have the ability to break/reform and reshuffle either autonomously or upon a suitable stimulus, thus endowing polymers with unprecedented properties, such as shape-memory, self-healing [8,11,16,17,18,19,20,21,22,23,24,25,26], recyclability [5,27,28,29,30,31,32], weldability [22,25,31,33,34,35,36,37,38,39], malleability, responsiveness to disparate stimuli, stress relaxation and (re)processability [4,13]. Historically, Lehn et al. [40] are acknowledged as the pioneers [41,42] of dynamic bonds, with the introduction of supramolecular chemistry. Supramolecular polymers rely on non-covalent reversible interactions, namely hydrogen bonding, metal–ligand coordination or π-π stacking [17,43] and have been proven intriguing for biomedical applications (e.g., supramolecular bioelastomers for drug delivery, cell co-culture and antibacterial surfaces [20]). However, the promptness of exchanges and instability to high temperatures made this new class of polymers appealing for softer smart materials in mild conditions, rather than advanced and high-performing thermosets/composites [4,13]. In the light of this limitation, a dynamic covalent network seems to be an essential feature for applicability in aerospace and automotive.

**Figure 2:**
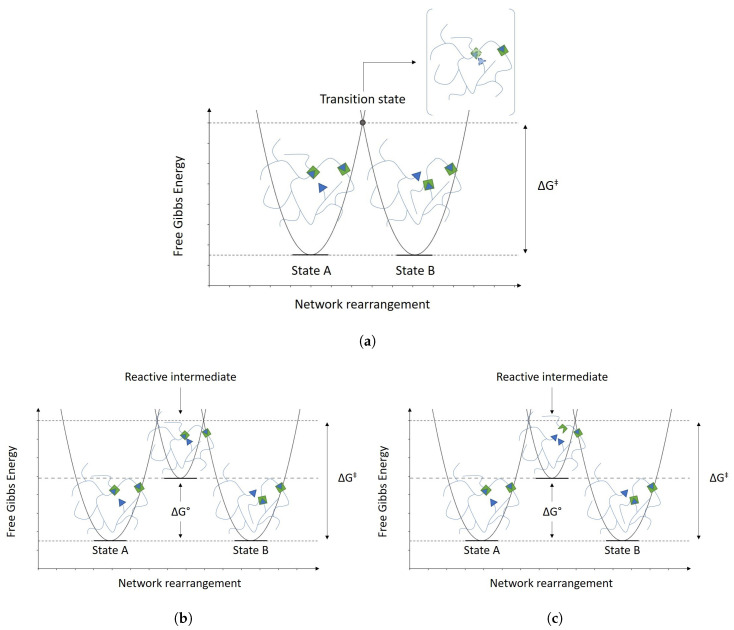
(**a**) Concerted molecular rearrangement: bond shuffling via unstable transition state. (**b**) Associative covalent adaptable network: bond forming/breaking pathway. (**c**) Dissociative covalent adaptable network: bond breaking/forming pathway. The arrows represent free moieties (e.g., –OH groups) that may attack the arrow-square complex either in the backbone or in cross-links (e.g., esters R-COO-R′). Sketch based on scientific considerations by Winne et al. in [3].

Dynamic Covalent Networks (DCNs), sometimes referred to as Covalent Adaptable Networks (CANs), may be the ideal option to address this task [4], as they rely on covalent interactions between repeating units. They could be either associative, following a bond forming/breaking pathway, or vice versa, dissociative with a bond breaking/forming mechanism. The differences, illustrated in Figure 2, go beyond mere chemistry-related speculations and are truly the ground that determines the kinetics of a smart polymer, as well as its mechanical and rheological properties. The mechanism in Figure 2a represents a concerted Molecular Network Rearrangement (MNR), wherein the transition between the initial State A and the final State B does not involve a reactive intermediate. Therefore, the transition state consists only of a temporary more cross-linked stage that is not associated with thermodynamic stability (drawn in square brackets in Figure 2a). The reactivity, and likewise the kinetics, depends exclusively on the overlapping of the free Gibbs energy curves for States A and B. This situation would be the ideal case scenario, as the cross-link density would be constant and there would be no possibility of side-reactions [3]. However, an actual MNR-featuring polymer would most likely show a complex multi-step pathway from State A to B. This is, of course, dependent on the chemistry of the interacting moieties, as well as the presence of a catalyst or external reagents. For the sake of a simple discussion, Figure 2b,c presents the case of only one reactive intermediate.

An associative CAN (Figure 2b) achieves bond exchange by stepping through an intermediate stage, whereupon cross-link density increases temporarily [3]. This effect is negligible, since the new bond is formed soon after the former is cleaved [3]. Therefore, the cross-link density can be considered approximately constant [3,4,6,7,13]. Contrarily, in the case of a dissociative pathway (Figure 2c), one ought to bear in mind some caveats. Dissociative CANs consist of bond breaking followed by bond forming with a temporary decrease in cross-link density, which causes a drop in viscosity and subsequent topology rearrangement [6,7]. As opposed to associative CANs, the loss of mechanical integrity and the reversibility of the reaction elicits a softening process, reminiscent of thermoplastic reprocessing [3]. In such systems, the preferred state (i.e., bonded vs. de-bonded) is the net result of two opposing driving forces: an entropic factor favouring de-cross-linking against an enthalpic push toward cross-linking [3]. Therefore, a sufficiently high temperature can shift the equilibrium toward the entropy-driven state, favouring debonding over bonding.

However, according to the particular chemistry at play, the temperature needed for such a shift could potentially exceed the degradation temperature of the network: this would be the case of a particularly high ΔH [3]. It is evident that this situation would entail an “apparently” retained cross-link density at all temperatures up to degradation (read as the low contribution of the de-cross-linked state). In other words, the line between associative and dissociative CANs could get quite blurry in some specific cases, wherein the dissociated state plays hardly any role at the observable temperatures [44]. Section 5.1 provides insight into examples of dissociative pathways and relevant applications thereof.

### 2.2. Vitrimers

Vitrimers have a characteristic temperature, namely the topology freezing transition temperature, T_v_, above which they flow with a predictable Arrhenius trend (analogous to silica-based glasses) [12,33]. This feature enables vitrimers to behave like a thermoset below T_v_ and like a viscoelastic re-shapable, malleable and re-processable fluid above T_v_ [3,4,6,7,8]. Although also thermoplastics are able to be reshaped, their change in viscosity over temperature is the consequence of a glass transition between two amorphous (or semicrystalline) regions, in agreement with the Williams–Landel–Ferry (WLF) theory [13,45]. Furthermore, thermoplastics can be eventually dissolved in a good solvent, if the temperature is high enough to shift the equilibrium toward the solution state [3]. Most importantly, in striking contrast with both thermoplastics and thermosets, vitrimers rearrange their network topology while retaining their cross-link density (read as mechanical integrity) and insolubility in solvents at all temperatures up to degradation [3,6,12,33,45]. In this sense, vitrimers proved to belong to a special class of polymers.

**Figure 3:**
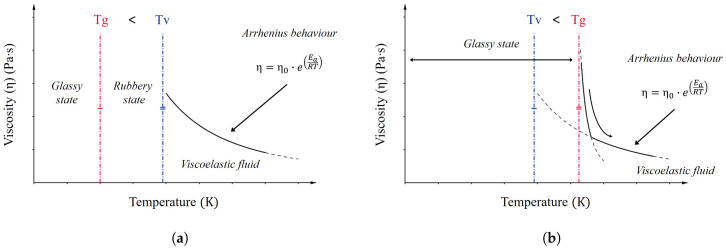
(**a**) Case with T_g_ lower than Tv. In this scenario, a glassy state and a rubbery state are distinguishable. (**b**) Case with T_v_ lower than T_g_. In this scenario, the flow becomes significant only above T_g_, and the dynamic cross-link rearrangement becomes eventually dominant. Further explanation provided in the text. Sketch based on considerations published by Denissen et al. in [6] and Van Zee et al. in [15].

In a way, topology freezing could be regarded as akin to a “second glass transition” [12], both because it is susceptible to the influence of the cooling rate and because of the observable third amorphous domain originating above T_v_ [45]. However, whereas T_g_ refers to the thermal onset of long-range coordinated chain motion between repeating units, the topology freezing represents the threshold above which dynamic molecular rearrangement through exchange reactions of cross-links becomes significant [6,14]. With respect to this, two possible cases can be discerned (see Figure 3).

Figure 3a shows a rather common case, that is when T_g_ is below T_v_. Firstly, the material transitions from a stiff and brittle glassy state to a soft rubbery state, with dramatic alterations of thermo-mechanical properties, as generally acknowledged in polymer science. Subsequently, as the temperature increases, the viscosity reaches 10^12^ Pa·s, conventionally chosen by Leibler et al. as the onset of topology rearrangement in their original study [12]. Eventually, when the time scale of material deformation is sufficiently larger than the time scale of bond reshuffling, the viscosity decays exponentially [6].

On the other hand, Figure 3b reports the case wherein the network is so inherently amenable to rearrange, that the activation energy for the exchange reaction is remarkably low. This results in the glass transition being the limiting factor for viscoelastic fluidity. Several surprisingly quick systems have been reported in the literature, such as the class of vinylogous acyls [46,47], fructose-derived bio-based polyimine vitrimers [42] or epoxy resins with exchangeable disulfide cross-links [28]. However, in such seldom cases, T_v_ must be regarded as a mere theoretical value, since it is extrapolated from stress relaxation plots in agreement with the Arrhenius law and Maxwell equation [6,12,14,28,42,46]. When T_v_ is estimated as lower than T_g_, the former would have no proper physical meaning, as the kinetics of molecular rearrangement is strongly hampered by the lack of free volume and frozen segmental motion [6,46]. As the system is heated up slightly above T_g_, the softening by activated segmental motion occurs in conjunction with exchange reactions until, eventually, the latter prevails (Figure 3b).

Unlike the case of T_g_, there is no general agreement on a protocol or experimental setup to assess T_v_. As mentioned above, the calculation customarily derives from the fitting of stress relaxation experiments performed at temperatures higher than T_v_ or, alternatively, from dilatometry (elongational expansion over time under a certain heat rate and low stress). Some other routes for gauging T_v_ have been pioneered, such as by means of aggregation-induced-emission luminogens or by thermomechanical analysis [14]. The latter hinges on a dimensional change as a result of activated exchange reactions in the proximity of T_v_ [14]; the former allows detecting a change in the fluorescence of luminogens-doped vitrimers [48]. Kaiser et al. reported a discrepancy between dilatometry and stress-relaxation, also as a consequence of the strong dependence of T_v_ on the applied stress and the heat rate [49]. They carried out elongational creep measurements by applying a series of stress values (10 kPa–200 kPa) and heat rates on different well-known catalysed epoxy/anhydride and epoxy/acids systems. They proved that low stress values lead to drawing deceptive conclusions on the actual kinetics of the system. In other words, as the applied stress increases, the T_v_ of the system settles to a minimum, which properly corresponds to the ability of the network to undergo bond exchanges [49]. This value effectively represents the onset of a rearrangement that is exclusively dependent on the chemistry of the system (equivalently, independent of the applied external load). Above this value, in the case of the softer epoxy/acids networks, the effect of the entropy-driven elastic recoil superimposes creep, thus shifting T_v_ again toward higher temperatures [49]. The shifting of T_v_ is less pronounced in the case of stiffer epoxy/anhydride networks. On a different note, the topology freezing transition temperature is also a direct consequence of parameters such as the cross-link density, inherent monomer mobility and stiffness and the exchange reaction kinetics (read as possible catalyst loading, favourable chemical affinity between moieties, thermodynamic factors) [6].

From all of the above, with the necessary caveats, one may sketch out the chief criteria that identify a vitrimer. Vitrimers stand out from dissociative networks and are more precisely classifiable as associative dynamic networks [3]. In their review [6], Denissen et al. suggested the following features as prerogative of vitrimers:

1. Organic network of covalently bound chains;
2. Thermally-induced topology rearrangement via associative exchange reactions (dynamic covalent network);
3. Above T_v_, viscosity follows the Arrhenius law and decreases as a consequence of the dominant kinetics of chemical exchanges.
4. Insolubility and cross-link density kept constant at all temperatures up to degradation.

As we pointed out in Section 2.1, some dissociative networks may exhibit all of the properties above; thus, the requirement of an associative network (point 4) is somehow wobbly and may be open to debate [3]. Another potential weakness of point 4 is the insolubility requirement: Breuillac and coworkers designed a polybutadiene vitrimer based on dioxaborolane chemistry, which involves the spontaneous and highly dynamic metathesis of boron-heterocyclic cross-links [50]. Due to the promptness and low activation energy of the dioxaborolane units [3,51], the network was fully dissolved in tetrahydrofuran after 120 h [15,50]. This proves that a prolonged exposure to a good solvent may ultimately lead to the dissolution of a vitrimer, if the conditions are such that they favour dynamic exchanges of bonds. As covered later, this offers a viable route for chemical recycling of vitrimeric matrices in composites (vide Section 4.2).

Having laid out the theoretical frame of the subject, Section 3 sheds light on the major hitherto proposed chemical strategies for dynamic covalent networks. Section 4 highlights some of the most enticing properties stemming from associative CANs and vitrimers. Finally, Section 5 brings to the reader’s attention some promising applications pertinent to all the systems mentioned.

## 3. Chemistry of Associative CANs and Vitrimers

As reported in many studies [6,10,28,51,52,53], the most attractive dynamic networks used for vitrimers and vitrimer-like materials rely on exchange reactions like transesterification, transamination of vinylogous acyls (urea/urethanes), transcarbamoylation, transalkylation of triazolium salts, disulfide exchange, imine-amine exchange, siloxane-silanol exchange and olefin metathesis. This section tackles the first four of the list (Section 3.1, Section 3.2, Section 3.3, Section 3.4). A final subsection presents the most typical catalysts and their role in the aforementioned reactions Section 3.5.

### 3.1. Transesterification

**Scheme 1:**
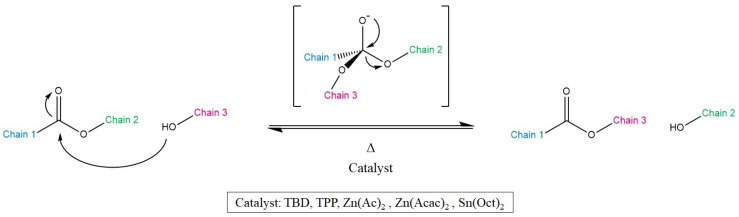
Representation of the Transesterification (TE) mechanism together with the most common catalysts. As a result, Chains 2 and 3 exchange positions, yet yielding again an alcohol and an ester.

As the name suggests, Transesterification (TE) is a well-known chemical reaction, wherein the organic group of an ester is exchanged with the group of an alcohol (Scheme 1). This reaction is typically catalysed by Brønsted acids [54,55], organo-metallic complexes or organic bases [14].

In 2011, Leibler et al. [12] paved the way to vitrimers with a TE-based resin, produced from the classical and well-established chemistry of epoxies. By reacting Diglycidyl Ether of Bisphenol A (DGEBA) with a mixture of fatty tricarboxylic and dicarboxylic acids (epoxy/COOH ratio 1:1) in the presence of Zn(Ac)_2_, they obtained an insoluble network able to flow and stress-relax at high temperatures. The stress-relaxation took several hours at 150 °C, and the activation energy was estimated to be 80 kJ mol^−1^ K^−1^, with a T_v_ as low as 53 °C for 10 mol% of catalyst. The reprocessability of this network was demonstrated by grinding the material into small bits and by performing injection moulding. Surprisingly, the original mechanical properties were fully restored. Motivated by these first results, in the same paper [12], the authors also tested a formulation based on the epoxy-anhydride system: glutaric anhydride (GA) and DGEBA (1:1 epoxy/acyl ratio) together with 5–10 mol% Zn(Acac)_2_. Even this system was successfully reprocessed via compression moulding and showed comparable activation energies with respect to the epoxy/acid system.

Since the publication of this study to these days, many other scientists were inspired to propose new ideas and applications for TE. As a matter of fact, epoxy resins based on TE could be very appealing for electronics, adhesives, coatings, light-emitting diodes or as matrices for composites [33]. Further strengthening the applicability of TE-epoxies, many attempts to implement them in advanced materials have been made. Just to mention some: silica-epoxy nanocomposites [52]; high T_g_ self-healing bio-epoxies from vanillin-guaiacol [22] or eugenol [5]; industrial rubbers and composites thereof [56]. The major strength of transesterification is that it is targeted to commercially-relevant and straight-forward chemicals, connoting a simple implementation. However, the major challenge is to meet the requirements for current thermoplastic processing techniques. The introduction of a simple thermoset resin able to readily flow in a few seconds and undergo several processing cycles would be in fact a revolutionary milestone in polymer engineering.

### 3.2. Transamination of Vinylogous Acyls

**Scheme 2:**
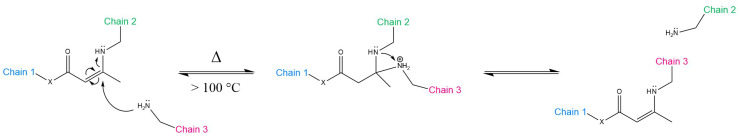
Mechanism of the transamination of vinylogous acyls. X = CH_2_, O, NR’R” corresponds to vinylogous amide, urethane and urea, respectively. The reaction can proceed without catalyst at 100 °C or above via the Michael addition pathway. The nucleophilic attack is performed by a free amine group. As a result, Chains 2 and 3 exchange positions.

Transamination of vinylogous acyls is a competitive alternative pathway to transesterification. As a matter of fact, the systems based on TE have typically the handicap of not being reprocessable fast enough for the industrial manufacturing standards (e.g., extrusion, injection moulding). The relatively slow stress relaxation leaves compression moulding as the only viable option [57]. Denissen et al. proposed two studies on both vinylogous urethanes [46] and vinylogous urea [47]. The general mechanism is illustrated in Scheme 2.

Because urethanes, ureas and amides are weaker electrophilic groups than esters, regular polyurethane, polyurea and polyamide networks are not prone to readily undergo bond exchange. Instead, the introduction of a vinylic bond inserted between the electron-donating nitrogen and the electron-withdrawing moiety confers Michael-type reactivity and strong resistance to hydrolysis [3,6]. As the name implies, transamination requires free amine groups to proceed, whose stability is crucial to prevent long-term oxidative damage [6]. Nevertheless, the straight-forward synthesis of polyurethanes/polyurea from acetoacetates/acetoamide and tertiary amines still represents an attractive path to explore [46,47]. The general trend that Denissen et al. observed in their studies is that the relaxation times decrease substantially as the “X” group becomes more electron-donating (amide → urethane → urea) [47]. Whereas vinylogous polyamides were discarded due to their sluggishness, vinylogous polyurethane relaxed in 85 s at 170 °C, and vinylogous polyurea with secondary amide functionality relaxed in only 57 s at the same temperature [47]. Adding a proton donor (a simple acid catalyst such as *para*-toluenesulfonic acid 0.5–6 mol%) accelerates the exchange reaction even further, therefore meeting the desired industrial standards for thermoplastic manufacturing [47,57]. In fact, vinylogous polyurea matrices were used for the production of glass fibre-reinforced vitrimers via pre-impregnation and thermoforming. The obtained six-ply laminates had a filler content of ca. 57% with E_11_ of about 40 GPa, comparable to a benchmark epoxy laminate for demanding applications [47]. In another study, Taplan et al. produced an acid-doped vinylogous polyurethane, which, quite remarkably, relaxed in 0.3 s at 160 °C [57]. The material readily lent itself to extrusion, performed at 150 °C with a double-twin screw extruder. The extrudate exhibited a defect-free finish with conserved mechanical and chemical integrity. The slight difference in stress relaxation may be ascribable to partial oxidation of free amine groups. As a final consideration, vinylogous acyls chemistry could definitely offer alternative materials for industry, although they currently suffer from a restricted chemistry and require care in the material preparation.

### 3.3. Transcarbamoylation

**Scheme 3:**
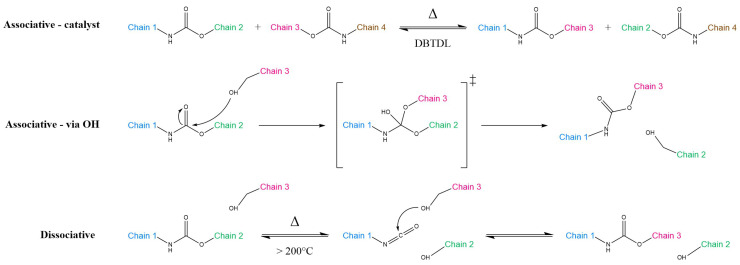
Transcarbamoylation of urethane units. In the associative-catalyst route (top), Dibutyltin Dilaurate (DBTDL) catalyses the reaction between two carbamate units. In the associative-via OH pathway (middle), a free –OH group performs a nucleophilic attack on the urethane. This is a viable route in polyhydroxyurethanes and resembles transesterification. Instead, in the dissociative mechanism (bottom), the urethane group first splits back into its original isocyanate and alcohol components, with temporary loss of cross-link density. Subsequently, it is able to react again with another –OH group, provided that hydrolysis does not take place first. In all of the cases above, Chains 2 and 3 eventually exchange positions.

Transcarbamoylation of carbamate groups in polyurethanes may take different pathways, depending on the chemical units involved (Scheme 3). The associative route could occur either via catalysed interaction between two distinct carbamate units or by nucleophilic addition of –OH groups, analogously to transesterification. On the other side, the dissociative route is strongly undesired, as it is often associated with detrimental side-reactions at high temperatures [58]. Because the carbamate group is less nucleophilic than esters, transcarbamoylation is notoriously sluggish [58,59,60,61], with much higher activation energies (*E_a_* = 99–159 kJ mol^−1^) when compared to transesterification (*E_a_* = 89–163 kJ mol^−1^) or transamination of vinylogous urethanes (*E_a_* = 31–129 kJ mol^−1^) [44]. For this reason, getting a sufficient rapidity would necessitate temperatures higher than 200 °C, thereby triggering the adverse reactions mentioned above. Thus, the applicability of transcarbamoylation for reprocessable polyurethanes remains a challenge.

Despite this, the doors to intrinsic plasticity in thermoset polyurethanes seem to be still open. Zheng et al. produced a polyurethane based on customary alcohol-diisocyanate chemistry, which is able to undergo comparably fast stress relaxation thanks to an organometallic catalyst (Dibutyltin Dilaurate (DBTDL)) [59]. Notably, the material is able to exhibit both standard elastic shape memory and thermal plasticity. In other words, above the activation temperature for bond exchanges, the network can be rearranged into a new desired permanent shape that is retained upon cooling. Conversely, slightly below the thermal threshold for transcarbamoylation, the thermoset can recover its original shape via the conventional shape memory effect [59]. The same authors improved their work by introducing a tetraol bearing tertiary amines in place of the catalyst [60].

A possible way to overcome the inherent limitations of traditional polyurethanes is to rethink their underlying chemistry. Polyhydroxyurethanes formed by multifunctional cyclic carbonate units and amines unlock the possibility to avoid toxic isocyanate species in the preparation, and they contain free –OH moieties for a supposedly faster bond exchange (Scheme 3, middle) [4,58]. Here, transcarbamoylation may proceed without catalyst and benefits from the concomitance of mechanical and chemical activation [58,62]. On top of that, according to Chen et al., the “non-vitrimeric” coupling of associative transcarbamoylation and dissociative cyclic carbonate aminolysis can definitely boost reprocessability [61].

### 3.4. Transalkylation

**Figure 4:**
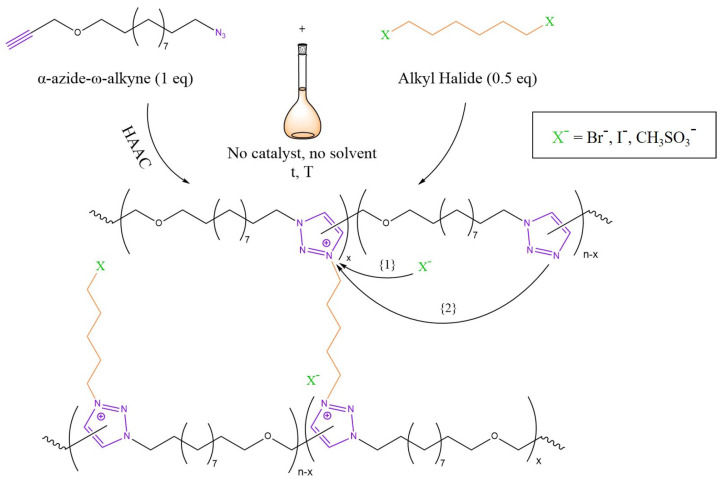
Mechanism of PTILs based on 1,2,3-triazolium salts. The Huisgen 1,3-dipolar Azide–Alkyne Cycloaddition (HAAC) condensation of an α-azide–ω-alkyne monomer takes place in concomitance with cross-linking by means of an alkyl halide. The reaction is one pot and requires no catalyst and no solvent. Possible counter-ions could be bromide (Br^−^), iodide (I^−^) or mesylate (CH_3_SO_3_^−^). The two possible bond exchange mechanisms are described in the text. Sketch based on the scientific work of Drockenmuller et al. in [53] and considerations from [3,6].

Transalkylation is an interesting mechanism that was employed in the design of ion-conductive vitrimers by Drockenmuller et al. in 2015 [53]. As summarised graphically in Figure 4, a Poly(1,2,3-Triazolium Ionic Liquid) (PTIL) network was synthesised by polyaddition of an α-azide–ω-alkyne monomer via Huisgen 1,3-dipolar Azide–Alkyne Cycloaddition (HAAC) and simultaneous cross-linking with an alkyl halide quaternising agent [53]. As highlighted in Figure 4, bond exchange may occur through two possible pathways. On the one hand, chain reshuffling could be initiated by a nucleophilic attack of the counter-ion on the alkyl-triazolium species (Arrow {1}) with a temporary de-cross-linking [3,6,53]. Such circumstances are reminiscent of a small particle-mediated dissociative pathway [3]. On the other hand, another theorised pathway may involve a concerted S_N_2-type nucleophilic addition mediated by a free triazole unit (Arrow {2}) [3,6,13].

Therefore, PTILs based on 1,2,3-triazolium salts may be regarded as the missing link between vitrimers and dissociative CANs [44]. The relaxation times of the obtained polymers ranged from 30 min at 130 °C to a few seconds at 200 °C [53]. Most interestingly, this seems to be a consequence of the counter-ion choice where Br^−^ leads to faster relaxation when compared to I^−^ or mesylate [6]. The low viscosity allowed reprocessing via powder compression moulding at 160 °C for 1 h. However, the mechanical properties deteriorated, possibly as a consequence of undesired-side reactions and chain scission. In spite of this limitation, this chemistry seems to be very promising for polyelectrolyte membranes for CO_2_ recovery and fuel cells or for super-capacitors and batteries as well [53]. Moreover, transalkylation chemistry based instead on sulfonium-sulphide could be promising for sulphur-cured rubbers [63].

### 3.5. Common Catalysts and the Role Thereof

**Figure 5:**
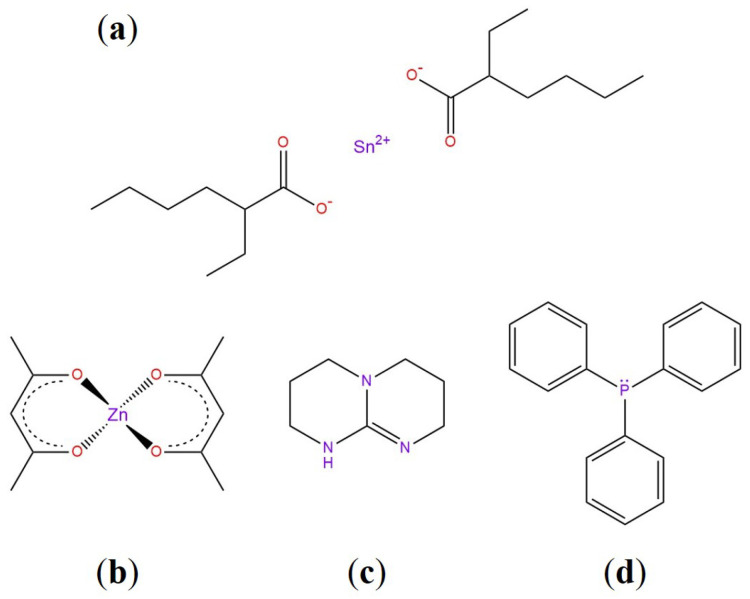
(**a**) Tin (II) 2-ethylhexanoate, Stannous Octoate (Sn(Oct)_2_). (**b**) Zinc acetylacetonate (Zn(Acac)_2_). (**c**) Triazabicyclodecene (TBD). (**d**) Triphenylphosphine (TPP).

**Figure 6:**
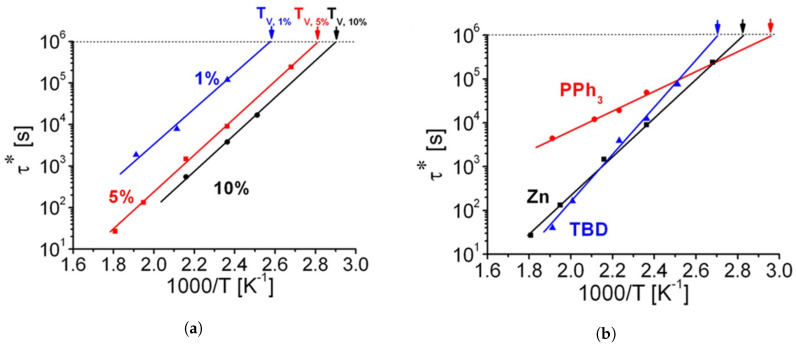
(**a**) Arrhenius plot of the measured relaxation times for 1, 5, and 10 mol% of Zn(Ac)_2_. As the concentration increases, T_v_ shifts to lower temperatures, and the relaxation time is shorter. The activation energy (slope of the curve) is dependent on the system, and therefore, it stays unchanged. (**b**) Arrhenius plot of the relaxation times for Zn(Ac)_2_, TPP (labelled as PPh_3_ by the original authors) and TBD at 5 mol%, measured from shear stress relaxation experiments. It is evident that the system containing TPP has lower T_v_, and it shows a lower reactivity, whereas TBD and Zn(Ac)_2_ have a comparable performance. Adapted with permission from [45]. Copyright 2012©, American Chemical Society, ACS.

The choice of a suitable catalyst is undoubtedly linked to the specific system at play. For transesterification, many studies prove the efficiency of organometallic complexes (tin or zinc organic salts) or bases such as Triazabicyclodecene (TBD) and Triphenylphosphine (TPP) [14,45,64]. 1-Methylimidazole was implemented for epoxy-acid systems as well [65]. The main chemicals we are going to cover in this review are illustrated in Figure 5. For vitrimers in general, the main rationale for using a catalyst is to enhance the bond exchange rate, thereby speeding stress relaxation up and tuning the topology freezing transition broadness (see Figure 6a) [45]. Naturally, the downsides would be a more complicated and more costly formulation, as well as the need to guarantee a homogeneous mixture. Several patents regarding vitrimer + catalyst formulations have been published, indicative of the strong potential applicability of vitrimers in the industrial sector [36,37,66].

**Zinc Acetylacetonate (Zn(Acac)_2_).** Thanks to its high thermal resistance, Zn(Acac)_2_ and similar zinc-based organometallic catalysts became the golden standard for transesterification chemistry, as reported in a plethora of studies [12,26,32,33,52,67]. The main role of zinc catalysts is threefold: (1) bring the reactive species together through directional coordination bonds, (2) increase the electrophilicity of the carbonyl group in esters by polarisation and (3) shift the alcohol/alkoxide equilibrium toward the more nucleophile alkoxide [68]. All these actions underlie β-hydroxy ester formation and transesterification [69]. A clever route was also explored by Niu et al. in a recent paper [70], where they produced a random copolymer of zinc methacrylate and acrylonitrile as a catalyst for DGEBA and sebacic acid. They demonstrated that the catalyst is more efficient than TBD and conventional Zn(Ac)_2_ under the same loading content (only ~0.75 mol%).

**Triazabicyclodecene (TBD).** TBD is a strong guanidine base that found application in vitrimer-like elastomers from nitrile-butadiene rubber [56], graphene-vitrimer composites [71] and photo-weldable carbon nanotube-reinforced vitrimeric matrices [35]. TBD is able to enhance the nucleophilicity of the alcoholic group via H-bond, and therein lies its high efficiency as a transesterification catalyst [72].

**Triphenylphosphine (TPP).** TPP can actively catalyse transesterification due to the lone pair available on its phosphorous atom [73]. When compared to Zn(Acac)_2_ and TBD, it has a lower activation energy and lower T_v_, promoting an overall more sluggish bond exchange rate (Figure 6b) [45]. To the best of our knowledge, the potential of TPP in vitrimers remains mostly unexplored.

**Stannous Octoate (Sn(Oct)_2_).** Brutman et al. designed a polylactide vitrimeric network, cross-linked with a diisocyanate in the presence of Sn(Oct)_2_ [74]. The catalyst is known for its catalytic activity in transesterification, high stability and low toxicity [74]. Crucially, the network rearrangement could occur even in the presence of a low number of free –OH groups. The delicate balance between catalyst load and the free isocyanate:OH ratio could significantly affect the properties of the network.

**Scheme 4:**
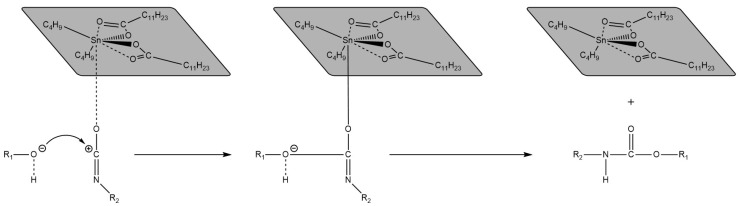
Illustration of the plausible mechanism of dibutyltin dilaurate as a catalyst for the cross-linking and transcarbamoylation of polyurethanes. Sketch based on considerations from [75].

**Dibutyltin Dilaurate (DBTDL).** Lastly, dibutyltin dilaurate, as mentioned in Section 3.3, has been used for transcarbamoylation. The polarisation of the isocyanate moiety facilitates the reaction with the alcohol [75], which can be useful both for catalysing cross-linking and transcarbamoylation (Scheme 4). A study suggested that DBTDL may also be useful for transesterification of epoxy-anhydride systems [76].

For alternative chemistries, one may want to deviate from the well-established catalysts found in literature. Snyder et al. reported the synthesis of polycarbonate vitrimers undergoing transcarbonation [77]. Polycarbonate has a great potential for sustainability, and it is massively employed (up to three million tons per year) for resistant and high-impact windows and safety glasses [77]. Transcarbonation occurs, mutatis mutandis, analogously to transesterification, with carbonate units reshuffling upon reaction with free –OH groups. Snyder et al. proved that Titanium (IV) isopropoxide (Ti(O*i*-Pr)_4_) successfully catalyses a vitrimer from 1,4-butanediol and bis-cyclic carbonate (activation energies as low as 81 ± 3 kJ mol^−1^ and relaxation times as low as τ* = 200 s for 4 mol% of catalyst) [77]. In addition to that, the combination of a Ti(O*i*-Pr)_4_-catalysed polycarbonate vitrimer matrix and natural cellulose has been proven appealing for novel green composites [78]. As a side note, Hatano and coworkers recently endeavoured to introduce a metal-free transesterification catalyst based on tetramethylammonium methyl carbonate [79].

## 4. Unique Properties of CANs

This section focuses on the most appealing properties derived from vitrimer chemistry: self-healing, recyclability and weldability. Based on our expertise, we claim that those are the crucial characteristics toward which the attention for vitrimers in industry (Section 5) is attracted.

### 4.1. Self-Healing

Unsurprisingly, the age-old interest in self-healing materials spans multiple industrial sectors, ranging from biomedical and biomimetics to protective coatings and aerospace [11]. The idea of increasing the lifespan of cutting-edge components by facile, autonomous or partially-assisted healing is indeed attractive. The first steps toward this goal were moved starting from defect-filling effects: the crack propagating in a material may rupture some microcapsules dispersed in it, causing the flood of an unreacted healing agent directly in situ [21]. This strategy is definitely intuitive and straight-forward, though it lacks repeatability, relies on a homogeneous dispersion of intact capsules, and requires attention to preserve the healing monomer inside them. Another study presented a way to harness maleimide chemistry for self-healing via crack filling in cold-curing resins [18]. Using a healing mixture of an aromatic bis-maleimide and a tetrathiol for a standard commercial amine-cured epoxy (i.e., EPON 828, equimolar conditions) yielded a healing efficiency as high as 80% after five days at room temperature. The maleimide functionalities covalently bind unreacted amines available at the crack surface and are subsequently prone to be cross-linked with the tetrathiol. Although the authors extended their work to large wind turbine blades produced via Reaction Injection Moulding (RIM), they judiciously highlighted that the healing agent may have a short shelf-life and may need encapsulation for a more solid applicability [18]. For the sake of completeness, we claim it is worth reporting Shape Memory Assisted Self-Healing (SMASH). It found application in both protective coatings for corrosion [21,24] and vitrimers made out of DGEBA and tricarboxylic acids or blends thereof [8,65]. The elastic recoil coming from shape memory effect is able to push the ruptured surfaces together, thus enhancing the healing process.

**Figure 7:**
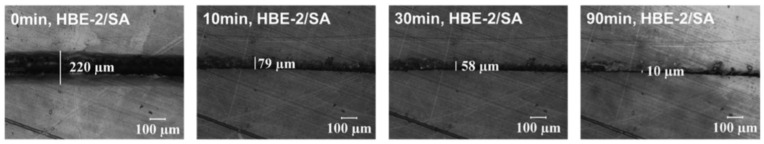
Optical micrographs showing the progressive healing of the hyperbranched polymer composite obtained from DER 331, 1,1,1-trimethylolpropane and succinic anhydride. The coatings were scratched and healed at 150 °C. Adapted with permission from [25]. Copyright 2018©, American Chemical Society, ACS. HBE, Hyperbranched Epoxy; SA, Succinic Anhydride; DER 331 is a commercial liquid epoxy resin.

**Figure 8:**
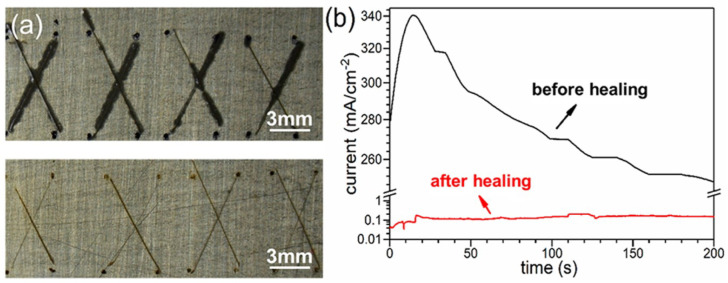
An example of an industrially-relevant application for self-healing coatings. The healing efficiency of the hyperbranched epoxy composite after 1 h at 150 °C entails a drastic reduction of the current density on a tin plate. (**a**) Corrosion of scratched (top) and self-healed coating (bottom). (**b**) Current density measured on the coated tin plate immersed in a 5 wt% NaCl solution before and after healing. Reproduced with permission from [25]. Copyright 2018©, American Chemical Society, ACS.

Ideally, a self-repairing material should be able to withstand autonomously an infinite number of healing cycles with no loss of properties or consistency. The fitting advent of CANs opened the doors to inherently healable and adaptable materials, which often require only a minimal external intervention via either temperature or light [6,13,26]. Both visible and UV light have been widely coupled with CANs subject to disulfide chemistry, in the same fashion as photoinitated RAFT polymerisation [16,17,80]. Reported protocols for an efficient healing of two disjointed pieces include: 8 h irradiation under N_2_ at 330 nm [16] and 24 h in air under visible light [17]. Michal et al. were able to heal a small scratch ( 150 μm) on a polydisulfide network in 5 min upon UV light (320–390 nm) irradiation [43]. Photostimulation is extremely potent, owing to its versatility, exquisite spatial and temporal control and contactless transfer of energy on a rather restricted area even at room temperature [16,81]. However, the majority of commercially available epoxies would rather benefit from thermally-activated reactions such as transesterification [14]. Han et al. developed an epoxy coating based on Succinic Anhydride (SA) and the hyperbranching of a commercial liquid resin (DER 331) (epoxy/anhydride ratio fixed at 1:0.5) [14,25,26]. The abundance of free hydroxyl groups deriving from branching DGEBA with a trifunctional alcohol allows both a catalyst-free curing and transesterification [25,26]. In their first work, it took only 90 min for the coating to self-repair a scratch from 220 μm to 10 μm at 150 °C [25]. The results are shown in Figure 7 and Figure 8 below. On top of that, a 80 μm thick coating robustly shielded a tin plate in a 5 wt% NaCl aqueous solution for 48 h. Because of its self-healing properties, the protection layer could still act as a barrier to corrosion after being scratched and healed again at 150 °C for 1 h (current density as low as 0.12 mA cm^−2^) [25].

The material showed weldability properties as well. In a more recent work, the same authors expanded the concept to a low viscosity mixture of DGEBA and DER 331 with a more detailed anti-corrosion properties investigation [26]. Self-healing materials thus gleam with a prodigious potential for industrial applications.

### 4.2. Recyclability

Most certainly, the possibility of recycling a thermoset material would be a refreshing and sought-after characteristic for many industrial fields heavily based on high performance composite materials. Several approaches have been investigated, namely mechanical, chemical and thermal (via pyrolysis) recycling [27].

Mechanical recycling is arguably the most straight-forward approach, for it simply consists of grinding the original vitrimer to a fine powder and hot pressing it to the desired shape again. High pressures are required in order to fuse the particles together: a greater contact at high temperature entails a more efficient bond exchange. Ruiz de Luzuriaga et al. designed a system based on DGEBA and an aromatic disulfide hardener for a Fibre-Reinforced Polymer Composite (FRPC) [28]. Based on experimental evidence, they concluded that the powder deriving from the control (DGEBA and aromatic diamine) could not be recycled to new specimens for tensile testing (200 °C, 100 bar, 5 min). On the contrary, using an aromatic diamine containing reversible disulfide bonds allowed a complete treatment, and the samples exhibited full recovery of the original mechanical properties [28]. Nevertheless, it is quite clear that this strategy relies on costly steps (i.e., grinding and pressing at high temperatures), would not preserve the integrity of reinforcements in composites or encapsulated parts in electronics and would lead to partial degradation over many cycles [5,14]. If the FRPC were to be ground and reprocessed for instance via compression moulding, its performance would be so severely compromised that it could only serve for non-structural applications [28]. Therefore, the need for a new route arises.

**Figure 9:**
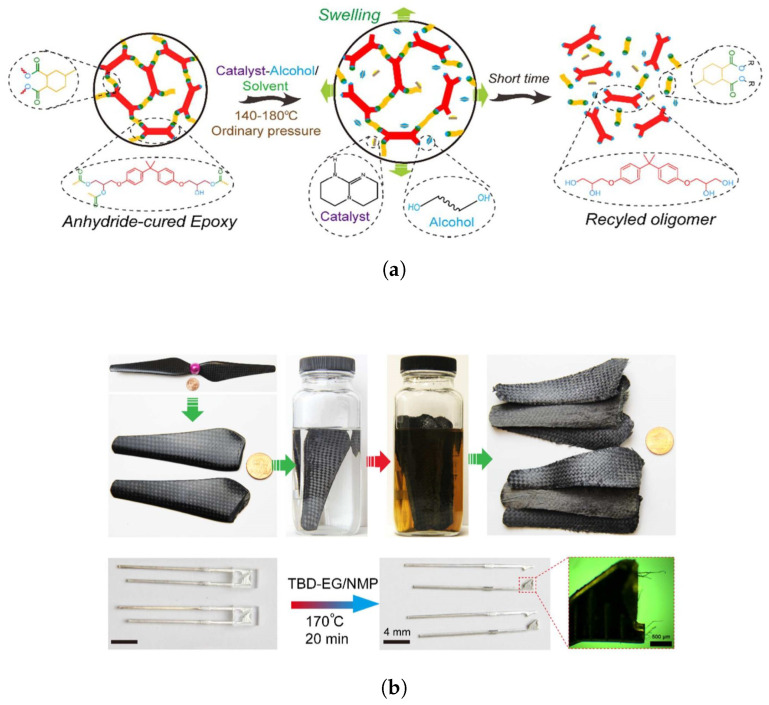
(**a**) Proposed route for chemical recycling of an anhydride-cured epoxy via swelling in an alcohol-based solution. (**b**) Applications of chemical recycling to composites: reclamation of carbon fibre fabric from a propeller after soaking in the recycling solution for 2 h at 170 °C (top); recovery of a metallic cathode from an epoxy-encapsulated LED (bottom). The metal was reclaimed undamaged. Both graphics are reproduced with permission from [27]. Copyright 2018©, American Chemical Society, ACS. EG, Ethylene Glycol.

Many studies we retrieved seem to be hinting at a chemical catalyst-assisted dissolution via a small molecule alcohol, such as Ethylene Glycol (EG) or ethanol, with a subsequent heating step aiming at the evaporation of the latter [5,27,28,29,30,31]. As presented in Section 4.3, this strategy may also yield promising welding. The general idea is that the solvent penetrates the network and takes part actively in transesterification, thus cleaving the chains into smaller fragments. The readily prepared solution is amenable to new curing as the solvent evaporates. In the case of composite materials, this is a straight-forward method to free the fibre mats from the matrix, in principle leaving them unscathed. In this regard, Kuang et al. prepared an FRPC based on an epoxy-anhydride matrix for a propeller [27]. They showed that a solution of TBD, NMP (*N*-Methyl-2-Pyrrolidone) and EG was able to separate the reinforcement from the vitrimer in only 2 h at 170 °C. The fibres were recovered in excellent conditions, as far as morphology and ultimate tensile strength were concerned. The feasibility of this protocol was further corroborated by successfully recovering the metal cathode of an LED from its epoxy capsule [27]. The metal surface showed no blemishes after the treatment. The results of this study are presented in Figure 9.

On another note, it has been proposed to use exchangeable bonds directly in the structural unit of the thermoset (in other words, in the backbone), to facilitate the formation of smaller fragments in dissolution [29]. Takahashi et al. envisioned a clever way to achieve this by cross-linking bis(4-glycidyloxyphenyl)disulfide (an epoxy showing a symmetric structure based on the S–S bond) with several diamines. By dissolving the matrix in diphenyl disulfide and a strong base for less than one hour, they showed via gel permeation chromatography that the network was indeed being fragmented into small chain segments. This route for a rapid and facile recycling process may offer potential for composites, yet it apparently relies on very specific chemical formulations. Aside from composites, it is worth noting that chemical dissolution is a viable option for 3D printing as well. Shi et al. used a DGEBA+fatty acids mixture as a recyclable ink for their components, achieving great consistency between each printing cycle [31]. The addition of Zn(Acac)_2_ and nanoclay particles was deemed beneficial for enhancing transesterification and the optimal shear-thinning properties. Their work focused on direct-ink-writing with the following protocol: pre-cross-linking under vacuum, 3D printing, pre-curing and full cure, recycling by full dissolution in excess of EG. Another study instead explored a diametrically opposite route that involves UV-curable reprocessable thermosets for high-resolution digital light processing [32]. The formulation consisted of an acrylate monomer, a diacrylate cross-linker, a zinc-based catalyst and a photoinitiator. The authors showed the repairability of the material both by reprinting a damaged figurine and by filling and curing with raw thermoset resin a circular hole in a printed strip [32]. In the former experiment, the figurine was fully refurbished after printing in situ and treating at 180 °C for 4 h; in the latter test, the flawed strip was filled with the formulation, irradiated for 10 min at 365 nm and heated up at 180 °C for 4 h to consolidate the boundaries via exchange reactions. Upon tensile testing, the sample fractured across its repaired circle, thus showing that integrity was achieved. Lastly, a printed sample could be successfully ground to powder (mechanical recycling) and moulded (500 MPa at 220 °C for 2 h) for the production of a non-structural part. Thus, Zhang et al. proposed a versatile solution to mitigate the environmental impact coming from the waxing consumption of materials for 3D printing [32]. The scarce mechanical properties may be boosted by reinforcement by means of nanoparticles or fibres.

A twofold recycling method was envisioned by Snyder et al. in their work on polycarbonate vitrimers [77] already mentioned in Section 3.5. The vitrimers recovered their original mechanical properties after being hot pressed at 160 °C (5–10 MPa). However, owing to the critical volatility of 1,4-butanediol used as a chain extender, the oxidation of the free –OH groups and the hydrolysis of the organometallic catalyst, the T_g_ decreased, and the process resulted in being lengthy and laborious. Alternatively, the same authors proposed a decarboxylation treatment in 1 M HCl at 90 °C for 36 h, in order to recover the original monomer for future uses. The extraction yielded 80 wt% of pure di(trimethylolpropane); however, butanediol was inseparable, and the titanium-based catalyst was hydrolysed to titanium oxide.

As a final consideration, one possible drawback of chemical recycling is the incomplete evaporation of the solvent, which may lead to decreased T_g_ [5]. In addition to that, progressive thermo-oxidation of the epoxy resin due to the high temperatures used, chemical waste and the lengthy procedure may reduce its attractiveness to industry.

### 4.3. Weldability

The ability to be welded (i.e., weldability) is the signature prerogative of metals, silica glasses and thermoplastics via chain reptation in the proximity of T_g_ [33]. Introducing this property to thermosets would be of utmost importance in many sectors, for it allows the manufacturing of complex shapes [33]. However, this is not easily done in practice, because thermosets can no longer be reprocessed once they undergo the irreversible curing reaction. With this in mind, Capelot et al. tried to test the properties of welded epoxy/acid systems in the presence of a metal catalyst, namely Zn(Ac)_2_ [33]. They showed that using 5 mol% catalyst and welding for 1 h at 150 °C under a 25% compressive strain produced the best mechanical properties for their samples. The amount of catalyst played a paramount role (i.e., helped transesterification and complete curing) for an effective welding, which was also corroborated by another study [34].

In some specific cases, light-assisted welding seems a more desirable option, for instance in the presence of complex geometries prone to warp or heat-sensitive components [35]. As covered above, this route has been implemented in UV light-responsive networks amenable to radical exchange: disulfide fragmentation reaction for poly-(ethylene glycol) hydrogels [80], UV photo-healing of elastomers based on trithiocarbonate under nitrogen atmosphere [16] and, lastly, an improved elastomer weldable by visible light in air thanks to thiuram disulfide chemistry [17]. However, the high reactivity of the radicals and the special character of the monomers and conditions used may prevent their applicability in industry [35]. In the light of the above, Yang and coworkers designed a photo-weldable vitrimer composite relying on the photothermal effect of multi-walled carbon nanotubes [35]. The vitrimeric matrix is based on DGEBA, TBD and adipic acid and is able to self-heal, be reshaped and welded via quick IR light irradiation, with excellent spatial and temporal control. The authors claimed that the strategy they introduced exhibited unmatched robustness, simplicity and potential for mass production [35].

Further options for the weldability of composite materials have been explored. Here, we mention a route reported by Charbert et al. for a long fibre composite with high filler concentration, manufactured via Resin Transfer Moulding (RTM) [34]. By applying pressures as high as 4.4 MPa, at 160 °C for 90 min, they achieved consistent force at break across multiple welding cycles. The protocol is promising for large-sized or hollow structures used in aeronautics. Nevertheless, there is still room for the optimisation of the curing process and matrix formulation, which could potentially mitigate time and cost overruns [34]. In fact, for most systems, it seems natural to assume that high temperatures and pressures up to hundreds or thousands of kPa are necessary [38]. However, when the network is extremely rigid, even 220 °C for 1 h may not be enough to produce nicely welded surfaces in a hot press [22].

Shi et al. envisaged a very interesting concept for solvent-assisted pressure-free welding of transesterification-based vitrimers [38]. They used the same mixture of DGEBA and fatty acids as the one Leibler and coworkers reported in their original work (vide Section 3.1), with the aid of Ethylene Glycol (EG) as a small-molecule, high boiling and OH-rich solvent. Owing to these properties, the solvent easily penetrates the interface that separates the materials and participates in the thermally-triggered transesterification during welding. The synergistic effect of bond exchange and diffusion of the solvent at the interface enables the cleavage and reforming of new strongly intertwined chains. After a first dissolution step in EG at 180 °C to elicit mild surface erosion, the samples were welded at the same temperature to ease evaporation of the excess solvent and, ultimately, welding [38]. This approach produced smooth and homogeneous welded surfaces with high fracture energies, and it could also be used to recycle vitrimers from ground debris with no need for external pressure. The same group elaborated a model to predict the elastic modulus and fracture energy of as-welded surfaces as a consequence of surface roughness, applied pressure and chain density evolution [39]. Lastly, they expanded the same solvent-assisted strategy to recyclable vitrimer inks for 3D printing [31].

## 5. State-of-the-Art of Industrially-Relevant Applications

### 5.1. Examples and Applications of dissociative CANs—Diels–Alder Reaction

**Scheme 5:**
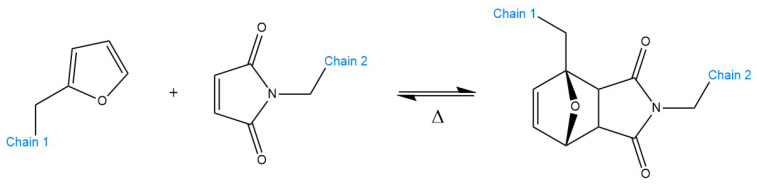
Schematic representation of a Diels–Alder reaction between a furan (left) and a maleimide (right) unit. When such functional groups are grafted on polymeric chains (Chains 1 and 2), they can enable reversible cross-linking. The reaction can revert back, typically, at 120–130 °C or above. The equilibrium constant Δ depends on the chemistry at play.

In this subsection, we present perhaps the most notable example of dissociative CAN chemistry with a promising scope in the industrial sector: Diels–Alder (DA) chemistry. The prototypical reaction, presented in Scheme 5, involves a [4+2] cycloaddition between an electron-rich diene and an electron-poor dienophile [4,13].

At elevated temperatures, the dynamic equilibrium can be shifted back toward the unbound state (retro-DA reaction) in a “click-like” fashion. A classical example of two interacting moieties are furan (diene) and maleimide (dienophile with electron-withdrawing ketone groups). Even though the DA cycloaddition is a fairly well-known process in chemistry (i.e., for cross-linking of polystyrene with furan-maleimide by Stevens and Jenkins in 1979 [4]), it was not until 2002, with the pioneering work of Wudl et al., that it gained popularity [4,13]. The network they obtained from tetrafunctional furan monomers and trifunctional maleimide units proved to be an appealing paradigm for sought-after properties such as shape memory, reprocessability and self-healing [4].

**Figure 10:**
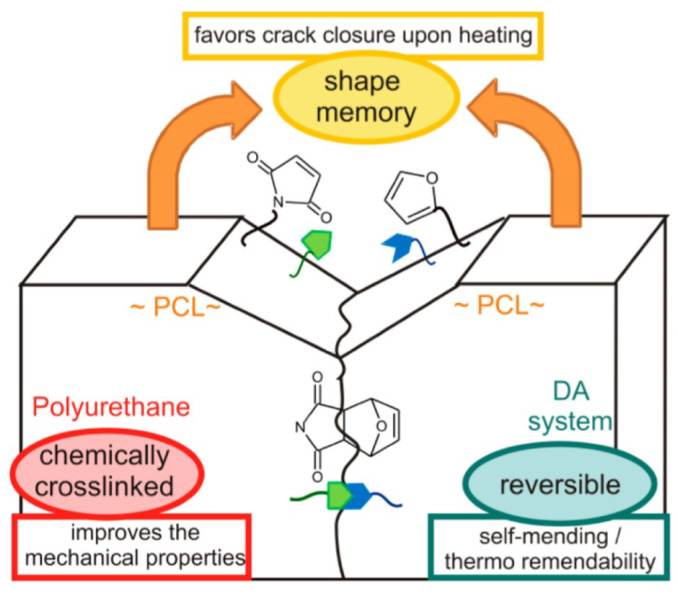
Schematic representation of crack closure as a synergistic effect of the shape memory effect and furan-maleimide chemistry in a PCL-based polyurethane. Reproduced with permission from [83]. Copyright 2014©, American Chemical Society, ACS.

The shape memory effect may be used to confer solid state plasticity on thermosets, whereby one can achieve the manipulation of complex geometry and shape memory multifunctional devices [82]. For instance, Rivero et al. designed a polyurethane network, wherein the shape memory property of polycaprolactone (PCL) units in the backbone assisted self-healing (SMASH) [83]. By applying heat, the synergistic effect of reversible furan-maleimide cross-linking units and elastic recoil favours rapid crack closure and network rearrangement Figure 10.

**Scheme 6:**
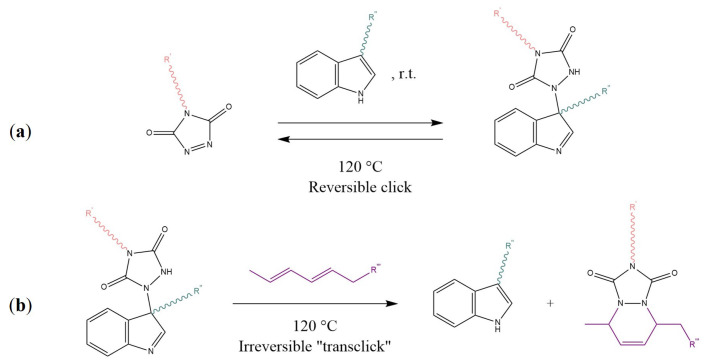
(**a**) Reaction between 1,2,4-Triazoline-3,5-Dione (TAD) and an indole at room temperature with a reversible click mechanism. (**b**) Irreversible transclick reaction between the TAD-indole adduct and a diene, with the formation of the original indole and a TAD-diene adduct. Sketch based on considerations from [84,85].

This “self-sewing” effect restores the original mechanical properties: the authors reported excellent healing of macro-scratches after one day at 50 °C [83]. Such behaviour is a function of the furan-maleimide groups density, as well as the content of hard segments (i.e., cross-link density, polycaprolactone segments) of the material. Another promising study presented by Billiet et al. introduced the concept of “click-transclick” chemistry applied to reversible Triazolinedione-indole (TAD-indole) Diels–Alder-like reactions [84]. The principle, presented here in Scheme 6, consists of a first click reaction between a TAD unit and indole, which proceeds spontaneously at room temperature with high yields and no need for a catalyst or any external stimuli (Scheme 6a) [84,85]. The now bound state can undergo an irreversible “transclick” reaction with a diene, thereby yielding the original indole and a new TAD-diene adduct (Scheme 6b). The authors applied this new concept to both a high T_g_ polymethyl methacrylate and a polyurethane, by means of a difunctional-TAD cross-linker [84].

**Figure 11:**
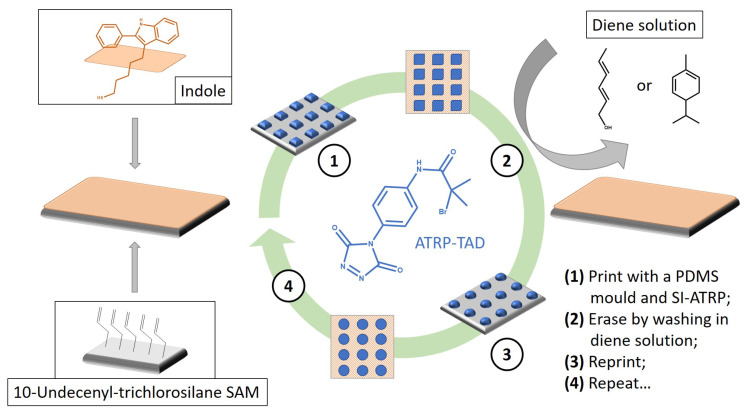
Schematic representation of the rewritable polymer brushes as per the original design of Roling et al. The explanation is available in the picture and in the text. SAM = Self-Assembled Monolayer. Sketch based on considerations from [85]. SI-ATRP, Surface-Induced Atom Transfer Radical Polymerisation.

The two networks could be reprocessed in a mould by extrusion and by compression, respectively. Recently, this knowledge stepped further from niche chemistry to concrete potential in nanostructured polymer substrates, useful in biosensing, information storage, electronics, and anti-fouling coatings [85]. Roling et al. designed some rewritable polymer brush micro-patterns [85], as represented in Figure 11. By preparing an indole-functionalised surface, they were able to graft a TAD-bearing Atom Transfer Radical Polymerisation (ATRP) initiator in a predefined pattern with a PDMS mould (①). By triggering the Surface-Induced reaction (SI-ATRP), they grew polymethacrylate micro-brushes. Subsequently, it was possible to reclaim the original indole surface by means of an erasing procedure via TAD-diene transclick reaction (e.g., with either a 2,4-hexadien1-ol or a -phellandrene solution) (②).

The quasi-pristine new surface was then amenable to multiple re-printing cycles with excellent spatial control (③ and ④). All of the studies mentioned above are clear examples of engineering materials where the dissociative nature of networks may be highly desirable.

### 5.2. Applications of Associative CANs and Vitrimers in the Industrial World

#### 5.2.1. Sustainable Vitrimers

With the soaring demand for environmentally-friendly materials and sustainable technology, researchers set out to harness bio-renewable sources. Quite interestingly, some studies demonstrated that vitrimers lend themselves to such purposes.

Lignin is the second most abundant polymer on Earth [86]. Most of the available technical lignin comes from residues of the paper making process [87], and represents a vastly underexploited source of biomass, with over 50 million tons produced per year [88]. Its polyphenolic structure seems to be promising for the formulation of alternative phenol-formaldehyde resins [87,89,90] and bio-epoxy asphalt [91], and it has been utilised in vitrimeric recoverable adhesives [86]. Nevertheless, owing to the low reactivity and low purity of the chemicals contained in lignin waste, some additional thermochemical treatments and costs are required (e.g., methylolation, phenolation) [87]. Therefore, the concrete inclusion of lignin in industry faces some practical hurdles. Zhang et al. prepared an adhesive based on catalyst-assisted transesterification between ozonated lignin and epoxidised sebacic acid [86]. For the highest lignin-to-epoxy ratio, the material showed the highest T_g_ (133 °C), fast relaxation (τ* = 81 s) and highest strength (12.9±1.5 MPa). More interestingly, the adhesive underwent a cohesive failure at 6.5 MPa when used to bond two aluminium sheets, which is comparable with commercial epoxy adhesives applied to join aluminium alloys [86]. Transesterification helped to achieve multiple bonding cycles with fairly satisfying consistency.

It is common knowledge in polymer engineering that Bisphenol A (BPA) has been deemed carcinogenic, toxic and endocrine-disrupting [92]. Therefore, the attention of industries is gradually shifting toward alternative epoxies for thermosets and composites. In the light of this, here we report studies on triglycidyl ether of phloroglucinol extracted from algae [92], polyimine networks from vanillin [93] or fructose [42] and derivatives of lignin [88]. In particular, diglycidyl ethers of vanillyl alcohol and bisguaiacol show great mobility, stiffness and thermal resistance (comparable to commercial BPA-based resins, such as EPON 828 and EPON 862) when cross-linked with a cycloaliphatic diamine [88].

**Figure 12:**
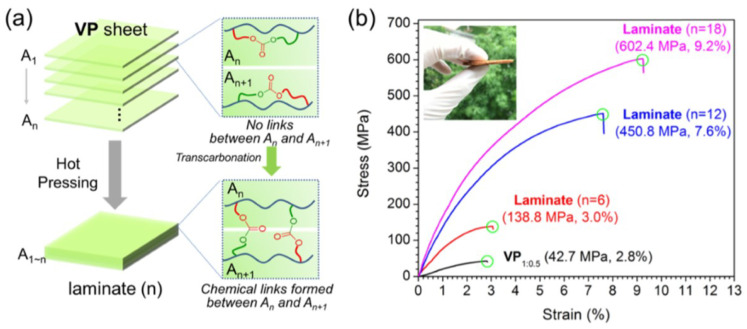
Demonstration of the reprocessing ability of curved Vitrimer-Paper (VP) composites using VP_1:0.5_ (bis-cyclic carbonate to propanediol ratio) sheets to prepare VP laminates via hot pressing. (**a**) Diagram of laminate preparation. (**b**) Tensile stress–strain curves of resultant laminates. Reproduced with permission from [78]. Copyright 2019©, American Chemical Society, ACS.

Cellulose is another abundant natural resource that has also found application in reinforced vitrimers, such as high water resistance nanopaper [64] and vitrimer-paper composites [78]. Zhao et al. focused on the latter application and proposed a transcarbonation-based polycarbonate matrix filled with a cellulose framework [78]. The dynamic nature of the matrix provided facile recyclability, reprocessability and self-healing. Besides, bond exchange in conjunction with hot pressing allowed the production of compact laminates with significantly improved mechanical properties (Figure 12). In addition to that, the authors soaked a criss-cross scratched vitrimer paper sheet in a solution of isopropyl alcohol containing Silver Nanowires (AgNW). The conductive fillers piled up preferentially into the notches. After healing the regions back at 160 °C, they obtained a foil with customised conductive pathways.

Another massively underutilised natural resource is crustacean shell waste, available to the extent of six to eight million tons per year [94]. Crab and lobster shells are made out of chitin (15–40%) [94], a polysaccharide that, along with its deacetylated derivative chitosan, has tremendous utility in biotechnology, environmental protection, pharmaceutics and nutrition [95,96]. Ghosh et al. developed a self-healing polyurethane based on an oxetane-substituted derivative of chitosan and a trifunctional isocyanate [97]. The network was able to self-heal in 30 min upon UV irradiation (280 to 400 nm) thanks to bond reshuffling enabled by the oxetane moiety. Lastly, vitrimer chemistry may be extremely useful for the production of green epoxidised-rubber composites. The release of toxic volatile organic compounds coming from the curing additives used in the rubber industry poses a serious hazard to health, as well as to the environment [67]. Zhang and coworkers synthesised a rubber via in situ epoxidation of an ethylene-propylene-diene monomer [67]. The cross-linking with bio-based decanedioic acid provided a matrix prone to undergo transesterification, which was then reinforced with carbon black. It was possible to recycle the composite via hot pressing a blend of rubber scrap with the uncured formulation. The shape memory effect, facile recycling with no need for grinding and non-toxic curing make this study a promising paradigm for green rubbers.

#### 5.2.2. Epoxy Resins

**Scheme 7:**
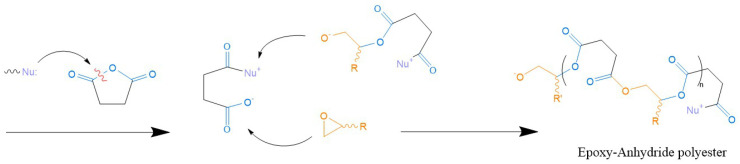
Mechanism of epoxy/anhydride curing (here with succinic anhydride). The reaction takes place via successive oxirane-anhydride ring opening with or without the aid of a catalyst. The final network could be regarded as a polyester/polyol.

**Epoxy/anhydride systems.** Using an anhydride as a hardener for epoxy thermosets is a rather popular industrial choice. The scope of this formulation includes, but is not limited to: LEDs, encapsulation of electronic parts, conductive ink paste and adhesives in structural components produced via RTM, where a high T_g_ is desirable [98]. The most plausible reaction mechanism (Scheme 7) consists of the alternating ring opening of the anhydride ring and oxirane ring (i.e., epoxide ring), resulting in a stiff polyester [33].

The reaction is strongly dependent on the initial nucleophilic attack, which can be performed by a catalyst or impurities such as water [99,100]. Whereas in the industrial practice, epoxy/anhydride ratios ranging from 1:0.8 to 1:1 are common, only ratios as low as 1:0.5 are able to guarantee a reasonable supply of free –OH groups for transesterification [5,22,33]. As usual, the trade-off between maximised stiffness and vitrimeric properties arises. Among the most popular anhydrides used for vitrimeric networks, we mention: succinic anhydride [5,25,26], glutaric anhydride [12,100], Methylhexahydrophthalic Anhydride (MHHPA) [22], phthalic anhydride for a high-performance recyclable epoxy [101], as well as many others like maleic or trimellitic anhydride mentioned in several patents [36,37]. A very recent study by Giebler et al. showed that it is still possible to achieve a high T_g_ (140 °C) in an uncatalysed formulation of aminoglycidyl resin and glutaric anhydride [100]. The tertiary amine present in the epoxy monomer is able to catalyse the transesterification in the network, yet at a slower rate than Zn(Acac)_2_. The use of a technically-relevant resin and a non-toxic hardener gives this concept some concrete potential for industrial applications.

**Scheme 8:**
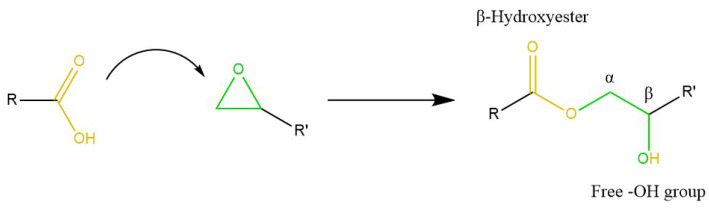
Mechanism of epoxy/carboxylic acid curing. Usually with the aid of a catalyst, the acid binds the open oxirane ring, resulting in a β-hydroxy ester with a free –OH group. The latter is amenable to act as a nucleophile to initiate transesterification.

**Epoxy/acid systems**. Epoxy/acid is the prototypical vitrimeric system, dating back to the original study by Leibler et al. in 2011 [12]. The reaction mechanism is not straight-forward due to some possible side-reactions. However, the addition of carboxylic acid groups to epoxy rings yields β-hydroxy esters in stoichiometric conditions (Scheme 8), which are the key factor in transesterification [12,33,45]. Formulations based on fatty acids and epoxy monomers are suitable for varnishes and coatings, and the produced esters impart good adhesion, flexibility and water resistance [102]. Tran et al. envisioned a strategy to minimise hydrolysis in an epoxy/acid vitrimer produced by “miniemulsion” polymerisation [69]. The process involves the formation of an emulsion of pre-compatibilised precursors, followed by cross-linking, drying and sintering. Although this complex route requires careful optimisation (i.e., by the addition of a surfactant to stabilise the colloidal dispersion), the low viscosity and delayed sintering kinetics of the produced material might open new possibilities for the production of paints, coatings, as well as composite materials via RIM or RTM [69]. Han and Xu doped a DGEBA-citric acid network with different contents of polymethyl methacrylate, thus producing a polymer-polymer composite [103]. Contents up to 10–25 wt% gave rise to the formation of a network structure in the vitrimer, thereby reaching an optimum in tensile strength while also shortening the relaxation time [103]. As a final note, we mention Pripol^®^ 1040, a mixture of di- and tri-carboxylic fatty acids used in combination with DGEBA in several studies focusing on vitrimers [31,52,65,68,104].

**Epoxies with disulfide chemistry.** The possibility of implementing a versatile chemistry such as disulfide chemistry in epoxies is definitely an intriguing option. In 1990, Tesoro and coworkers introduced an epoxy network cross-linked with a disulfide-containing diamine, showing the degradability and recyclability of the material by cleavage of S–S bonds [105]. By retracing these steps, another paper showed that a better degradability can be achieved by implementing disulfide bonds in the epoxy monomer instead [29]. Notably, disulfide metathesis can work in synergy with transesterification, as reported by Chen et al. in [106]. They introduced the concept of a dual vitrimer, where transesterification between DGEBA and dithiodibutyric acid is catalysed by TBD and disulfide exchange is triggered by heat. The material exhibited very fast stress relaxation (1.5 s at 200 °C with a very low estimated T_v_) and excellent reprocessability after four cycles at 100 °C (1 h each). Lastly, as we have already brought to the reader’s attention in Section 4.2, epoxies with disulfide chemistry may find applicability in FRPC [28].

#### 5.2.3. Elastomers

Vulcanised rubbers quite arguably take a large share of the industrial market for elastomers, with more than 27 tons produced yearly, which represents a considerable hazard for the environment [50,63]. The most common attempts to reuse scrap elastomers consist of either grinding rubber waste into powder or chemical desulphurisation to reclaim the pre-vulcanized rubber network [63,67]. However, the original properties are irretrievably lost, thus leaving this option acceptable only in the case of less critical applications with low requirements [63]. The interest in vitrimeric elastomers gravitates around these technological challenges. Here, we report studies on hydrogenated carboxylated nitrile-butadiene rubber [56], and composites thereof (study by our group under revision as of July 2020), polybutadiene/dioxaborolane networks for creep-resistant rubbers [50], carbon black-reinforced rubbers based on transesterification [67], recyclable sulphur-cured rubbers via transalkylation [63] and reprocessable epoxidised natural rubber via disulfide rearrangement [10].

Vitrimeric elastomers are also appealing for smart electronics, such as energy harvesters and tactile sensing elements [107]. Deng et al. proposed an elastomer amenable to disulfide exchange for encapsulating a conductive silver nanowire network [107]. The vitrimeric properties allowed for a facile and versatile fabrication of a triboelectric nanogenerator by multiple assembly of building blocks in a jigsaw puzzle-like fashion. The complex manipulation into freely-designable 3D structures with an origami strategy entails great potential for tactile sensors, with a possible extension to transistors in the foreseeable future [107].

Lastly, we report some studies on Polydimethylsiloxane (PDMS), a ubiquitous elastomer with potential in electronics (e.g., as an ion-selective membrane in Ion-Sensitive Field-Effect Transistor (ISFETs), spring material in accelerometers or in sensors with integrated electronics) [108]. Zheng and McCarthy revived the attention for siloxane equilibration as a “simple, robust and obvious” self-healing mechanism, possibly overlooked ever since the first publications from the 1950s [23]. They designed a living polymer network from the equilibrium of cyclosiloxane oligomers and tetramethylammonium dimethylsilanolate. Stability to hydrolysis, catalyst-free formulation and a low-cost monomer are the major advantages. Schmolke et al. tried to apply this concept to a room-temperature self-healing PDMS [11]. Lastly, Stukenbroeker and coworkers reported a quenchable PDMS vitrimeric network with dynamic vinylogous urethane cross-links [109]. Stress relaxation could be both activated thermally and suppressed on demand by reacting a -ketoester with the free -NH_2_ groups. This “masking” effect eventually impedes transamination, hence leading to a quenched state [109].

#### 5.2.4. Composites and Nanocomposites

As customary in industrial practice, polymeric matrices are usually reinforced by means of inorganic fillers to boost characteristics such as thermomechanical properties, wear performance and environmental resistance. When the filler comes in the form of small solid particles with dimensions below 100 nm, these materials are referred to as nanocomposites. Krishnakumar et al. used Graphene Oxide (GO) nanofillers in an epoxy vitrimer that underwent network rearrangement by disulfide exchange [110]. Intriguingly, a higher GO content generates additional free volume, thus lowering T_g_ and enhancing chain mobility. As a consequence of this, self-healing and shape memory properties are more favourable than in unfilled matrices. This effect, together with the improved flexural strength and modulus (+7.1% and +9.4% for 1 wt% GO, respectively), contributes to the attractiveness of the formulation [110].

However, this approach takes advantage of the poor interaction between GO and the organic matrix. On the contrary, a good adhesion between filler and matrix is desirable in composites, in order to assure a correct load transfer. Legrand and coworkers designed nanocomposites based on epoxy-functionalised silica nanoparticles [52]. The grafted chains limit filler aggregation by steric hindrance and favour a good dispersion state. Although unfunctionalised particles showed the tendency to constrain chain movement, the active β-hydroxy esters directly bonded onto the surface improve stress relaxation substantially [52]. The authors then proceeded with the production of a large batch of nanocomposite formulation (25 wt% of industrially-relevant silica particles), proving the flexibility of their design. In an unpublished study by our group (under revision as of July 2020), we proposed a similar approach for composites from Hydrogenated carboxylated Nitrile-Butadiene Rubber (HXNBR) and epoxy-functionalised calcium silicate particles (esilicate). The urge for rubber reinforcement with a high filler content rises from the poor mechanical performance of the raw elastomer, and it is therefore tremendously desired for industrial applications. In our unpublished study, we used the functionalised particles both as the only source of exchangeable bonds and as the cross-linker. Because of this, the TBD-catalysed transesterification became faster as the amount of the filler increased. This unusual advantage came together with a mild plasticising effect, which led to a decrease in T_g_ with the inclusion of more esilicate particles. The incomplete stress-relaxation makes this material more appropriately regarded as a vitrimer-like composite.

Another clever concept was proposed by Yang et al. in [71]. They produced a graphene-reinforced resin based on TBD, DGEBA and sebacic acid. The shape memory effect allowed switching from a 2D shape to a 3D one via a twofold approach: besides the standard thermally-triggered transesterification, graphene enabled energy conversion upon Near-Infrared Radiation (NIR). That is to say, graphene interacts with light to produce heat (which goes by the name of the photothermal effect), thus activating network rearrangement indirectly. This effect allowed shape recovery in roughly 1 min. Moreover, as the graphene loading stepped from 0% to 1 wt%, the material showed ductile fracture, with larger yield strength and strain at break [71]. The same dual-triggered approach was used by Yan et al. in a vitrimeric polyurethane reinforced with carbon-nanotubes [111]. The response to NIR enabled shape recovery and reshaping under heat and light with no mould, thanks to the accelerated transcarbamoylation.

On a different note, Yu et al. tried to improve the production protocol of compression moulding for long fibre composites, with the introduction of a vitrimeric powder [104]. The vitrimer particles become cured and sintered, while at the same time reinforced with a carbon fibre mat in one step. Since there is no precuring stage for the matrix, the procedure could be completed in only 1 min (1 MPa at 300 °C) [104]. The malleability was demonstrated by reshaping the produced sheet into a pinwheel and by subsequently heating up at 200 °C for 3 h. Other relevant studies on composites have been covered in the other sections above [27,28,34,35,78].

## 6. Outlook and Conclusions

In summary, our work offers a comprehensive overview of CANs as a potent and fresh resource in polymer science and engineering. With the increasing concern for environmental issues and hazardous chemical waste, the possibility of extending the service of thermosetting polymers via self-healing and recycling is undoubtedly intriguing. As presented above, this option is viable for applications in the aerospace, automotive, electronics and biomedical sectors, as far as composites, sustainable thermosets from bio-renewable sources, epoxies, elastomers and polyurethanes are concerned. Vitrimers, introduced to the scientific community no longer than nine years ago, are a budding class of thermosets with the ability to flow above a certain characteristic temperature determined by the chemistry involved. This property is achieved by covalent bond exchange, for instance via transesterification, transamination or disulfide metathesis. The underlying concept of vitrimers is based on the relatively simple idea of a network amenable to self-arrange upon stimulation, ideally with no loss of integrity and no undesired side-reactions over time. Therein lies their potential. However, the current dearth of practical applicability in industry may be ascribable to some inherent limitations. Firstly, to the best of our knowledge, the trade-off between self-healing (or recycling) efficiency and mechanical performance still represents a major scientific conundrum. Secondly, the typical long relaxation times observed in vitrimeric systems are far away from meeting the standards of conventional processing routes for thermoplastics, where the molten polymer is shaped in a few seconds. On the contrary, the applicability in composite processing seems much more promising, albeit still vastly underexplored. Further drawbacks are the lack of a well-established protocol for gauging T_v_, as well as the shortage of fit-for-purpose, sustainable formulations. In view of the above-mentioned challenges, we are thrilled to be contributing further to the improvement of vitrimers. We are confident that a bright future is ahead for the applicability of CANs in industry.

## Abbreviations

The following abbreviations are used in this manuscript:

DCN	Dynamic Covalent Network
CAN	Covalent Adaptable Network
MNR	Molecular Network Rearrangement
T_g_	Glass transition temperature
T_v_	Topology freezing transition temperature
TE	Transesterification
TBD	Triazabicyclodecene
TPP	Triphenylphosphine
Zn(Ac)_2_	Zinc Acetate
Zn(Acac)_2_	Zinc Acetylacetonate
Sn(Oct)_2_	Stannous Octoate
Ti(O*i*-Pr)_4_	Titanium (IV) Isopropoxide
DBTDL	Dibutyltin Dilaurate
DGEBA	Diglycidyl Ether of Bisphenol A
FRPC	Fibre-Reinforced Polymer Composite
RTM	Resin Transfer Moulding
RIM	Reaction Injection Moulding
SMASH	Shape Memory Assisted Self-Healing
DA	Diels–Alder (reaction)
PDMS	Polydimethylsiloxane
RAFT	Reversible Addition-Fragmentation chain Transfer
WLF	Williams–Landel–Ferry
PTIL	Poly(1,2,3-Triazolium Ionic Liquid)
GA	Glutaric Anhydride
EG	Ethylene Glycol
NMP	N-Methyl-2-Pyrrolidone
MHHPA	Methylhexahydrophthalic Anhydride
NIR	Near-Infrared Radiation
